# Anatomic repair and ligament bracing as an alternative treatment option for acute combined PCL injuries involving the posteromedial or posterolateral corner—results of a multicentre study

**DOI:** 10.1007/s00402-023-05015-5

**Published:** 2023-09-11

**Authors:** Tobias J. Gensior, Bastian Mester, Andrea Achtnich, Philipp W. Winkler, Ralf Henkelmann, Pierre Hepp, Richard Glaab, Matthias Krause, Karl-Heinz Frosch, Johannes Zellner, Christian Schoepp

**Affiliations:** 1 OPND Clinic Neuss-Düsseldorf, Neuss, Germany; 2 Department of Trauma, Hand and Reconstructive Surgery, University Hospital Essen, Essen, Germany; 3 Department of Sports Orthopaedics, Klinikum Rechts der Isar, Technical University of Munich, Munich, Germany; 4 Department of Orthopaedics and Traumatology, Kepler University Hospital Linz, Linz, Austria; 5 Department of Orthopedics, Trauma and Plastic Surgery, University of Leipzig, Leipzig, Germany; 6 Department of Traumatology, Cantonal Hospital Aarau, Aarau, Switzerland; 7 Department of Trauma and Orthopaedic Surgery, University Medical Center Hamburg-Eppendorf, Hamburg, Germany; 8 Department of Trauma Surgery, Orthopaedics and Sports Traumatology, BG Clinic Hamburg, Hamburg, Germany; 9 Sporthopaedicum Regensburg, Regensburg, Germany; 10 Clinic for Arthroscopic Surgery, Sports Traumatology and Sports Medicine, BG Clinic Duisburg, Duisburg, Germany; 11 Trauma Committee of the AGA (Society for Arthroscopy and Joint Surgery), Zurich, Switzerland; 12 Ligament Committee of the AGA (Society for Arthroscopy and Joint Surgery), Zurich, Switzerland

**Keywords:** Combined PCL injury, Posteromedial corner, Posterolateral corner, Ligament bracing, Augmented suture repair

## Abstract

**Introduction:**

Combined PCL injuries involving the posteromedial/-lateral corner (PMC/PLC) usually require surgical management. Literature shows controversy regarding the standards of treatment. Suture-augmented repair leads to excellent results in acute knee dislocations but has not been investigated clinically in combined PCL injuries. The purpose of this multicentre study was to evaluate the clinical outcome of this technique in acute combined PCL injuries.

**Materials & methods:**

N = 33 patients with acute combined PCL injuries involving the PMC/PLC were treated by one-stage suture repair with ligament bracing of the PCL and suture repair of the accompanying PMC/PLC injuries with/without ligament bracing or primary augmentation by semitendinosus autograft. Outcome was assessed by IKDC questionnaire, Lysholm Score, Tegner Activity Scale and KOOS. Additional PCL stress-radiography was performed.

**Results:**

N = 31 patients with combined PCL injuries (female: male = 7:24; age 39.1 ± 13.8 years) with a follow-up of 16.8 ± 9.6 months were finally evaluated. 18 had PMC injuries, 13 PLC injuries. 32.2% presented with accompanying meniscal tears (70% medial meniscus). 19.4% showed cartilage injuries grade III-IV. Complications included one infection and four knee stiffnesses. Three had symptomatic postoperative instability, all affiliated to the PLC group. The IKDC was 69.8 ± 16.5, Lysholm score 85 ± 14.4 and KOOS 89.7 ± 8.1. Median loss of activity (Tegner) was 0.89 ± 1.31. Comparing PMC and PLC, all scores showed a tendency towards more favourable outcomes in the PMC group (n.s.). Stress-radiography showed an overall side-to-side difference of 3.7 ± 3.8 mm. Subgroup evaluation showed statistically significant better results (p = 0.035) of PMC (2.5 ± 1.5 mm) versus PLC (5.8 ± 5.6 mm).

**Conclusions:**

One-stage suture repair with ligament bracing is a viable technique for acute combined PCL injuries and predominantly leads to good and excellent clinical outcomes. Patients with PLC injuries show a tendency towards inferior outcomes and higher instability rates compared to PMC injuries. These results may help in therapy planning and counselling patients with these rare injury pattern.

**Level of evidence:**

Level II.

## Introduction

The correct management of injuries to the posterior cruciate ligament (PCL) remains controversial. Non-operative treatment is generally accepted for isolated PCL tears due to its excellent intrinsic healing potential and good functional outcome [1]. Surgical management is recommended in acute cases with additional peripheral ligamentous or intraarticular injuries, or chronic symptomatic instability. [2–4]. The grade of instability is increased significantly in combined injuries involving the collateral ligament complexes [5], leading to a functional instability with higher loading forces to the joint cartilage and a higher risk of posttraumatic arthritis of the knee [6–8]. Therefore, combined PCL injuries as well as knee dislocations should preferably be managed operatively [9, 10].

Despite available data concerning the management of isolated collateral ligament injuries, there is controversy regarding the ideal technique of treatment of acute combined PCL injuries involving the posteromedial or posterolateral corner (PMC/PLC) of the knee. Evidence is weak in terms of recommendations whether to repair torn structures by suturing with or without ligament bracing versus primary ligament reconstruction.

In 2014, a new technique of single-stage augmented primary suture repair in multiligamentous knee injuries by ligament bracing was introduced by Heitmann et al. [11]. The same working group could demonstrate a higher loading endurance in comparison to both ligament reconstruction and isolated suture repair [12]. Finally, the results of a prospective multicentre trial revealed predominantly good to excellent clinical results after augmented primary suture repair in multiligamentous knee injuries [13].

It has to be considered that these investigations as well as studies of two other study groups focused on the treatment of multiligamentous injuries in the context of knee dislocations [14–16].

In current literature, the surgical principles of refixation and ligament bracing of isolated PCL tears are mostly described in case reports and technical notes [17–24]. Though, combined PCL injuries involving the PMC or PLC are neither mentioned as a separate entity nor investigated in a larger patient population.

The aim of this multicentre study is to evaluate the clinical outcome after surgical repair with additional suture augmentation by ligament bracing in acute combined PCL injuries. It was hypothesized that an augmented one-stage suture repair would lead to similar good clinical and radiological results as recently seen in the treatment of knee dislocations. To our knowledge, up to now this is the first investigation to evaluate the outcome of this technique clinically for combined PCL injuries.

## Materials and methods

For this study, ethical approval was obtained initially from the ethical committee of the Ärztekammer Nordrhein (Düsseldorf, Germany) responsible for the leading study centre (no. 2019159), and then separately from the local ethical committees of all participating trauma centres.

Between 2016 and 2019, n = 33 patients with acute combined PCL injuries were treated with one-stage suture repair and ligament bracing of the PCL and repair ± ligament augmentation of the PMC/PLC injuries. As two patients were lost to follow-up, n = 31 could be included in this investigation. All patients were operated within 14 days at six trauma centres in Germany (five centres) and Switzerland (one centre). The responsible local leaders of the contributing departments of this multicentre study are affiliated to the AGA Trauma and Ligament Committees of the German-speaking Society for Arthroscopy and Joint Surgery (AGA, Switzerland).

Patients with clinical and radiological evidence of acute PCL tears in combination with injuries of the PMC or PLC were included. In advance, patients were informed meticulously about purpose and operationalization of the study and agreed to participate.

**Fig. 1:**
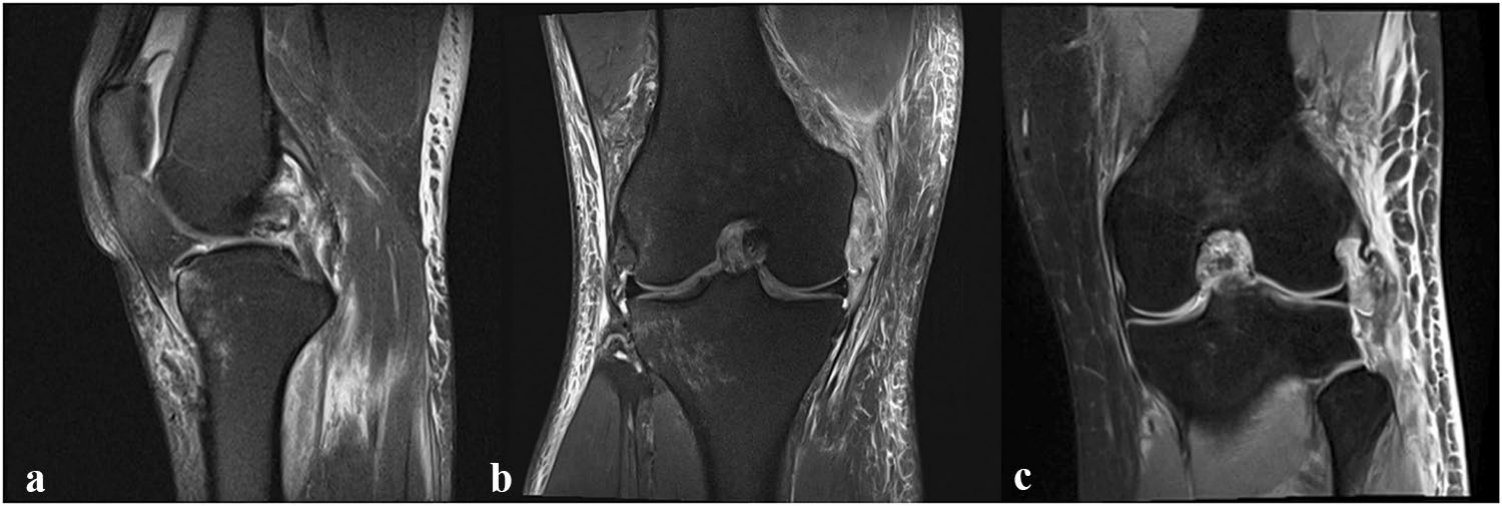
Preoperative MRI of a combined PCL lesion with proof of intraligamentous PCL tear (**a**) and either PMC injury with femoral MCL avulsion (**b**) or PLC injury with fibular LCL avulsion (**c**). *PCL* posterior cruciate ligament; *PMC* posteromedial corner; *PLC* posterolateral corner. *MCL* medial collateral ligament. *LCL* lateral collateral ligament

All patients were categorized grade II and grade III PCL injuries according to the classification by Cooper et al. [25]. Indications for surgical treatment were supported by clinical examination and recent MRI, which was available for all patients (Fig. 1). The maximum interval between injury and operation was set to 14 days, derived from the previous experiences in acute knee dislocations [13]. Early surgical intervention was demonstrated to be beneficial due to scarring and quick retraction of ligamentous structures [26].

Exclusion criteria were patient age younger than 18 years, open growth plates as well as patients with combined PMC plus PLC injuries, additional ACL injuries and knee dislocations. Furthermore, patients suffering from chain injuries of the same extremity, polytrauma, pelvic injury, popliteal artery dissection and patients treated with two-stage procedures were not suitable for inclusion into this study.

The diagnosis derived from clinical examination, stress-radiographs and MRI was secured by examination-under-anaesthesia of the injured knee and arthroscopic evaluation.

### Surgical procedure and rehabilitation

The surgical strategy includes diagnostic arthroscopy and treatment of concomitant intraarticular injuries, arthroscopic primary PCL suture repair and PCL bracing as well as suture repair/refixation of the injured peripheral structures with or without additional ligament bracing or alternatively primary ligament augmentation by semitendinosus autograft.

Anaesthesia as well as peri- and postoperative pain management are conducted according to the standardized protocols of each participating trauma centre. Patients are operated in supine position and by the use of an electric or mechanical leg holder. At first, diagnostic arthroscopy is done to confirm the diagnosis and address additional intraarticular pathologies. Accompanying meniscal tears are treated preferably by meniscal suturing, alternatively by partial meniscectomy. If present, chondral injuries are addressed by debridement and microdrilling.

**Fig. 2:**
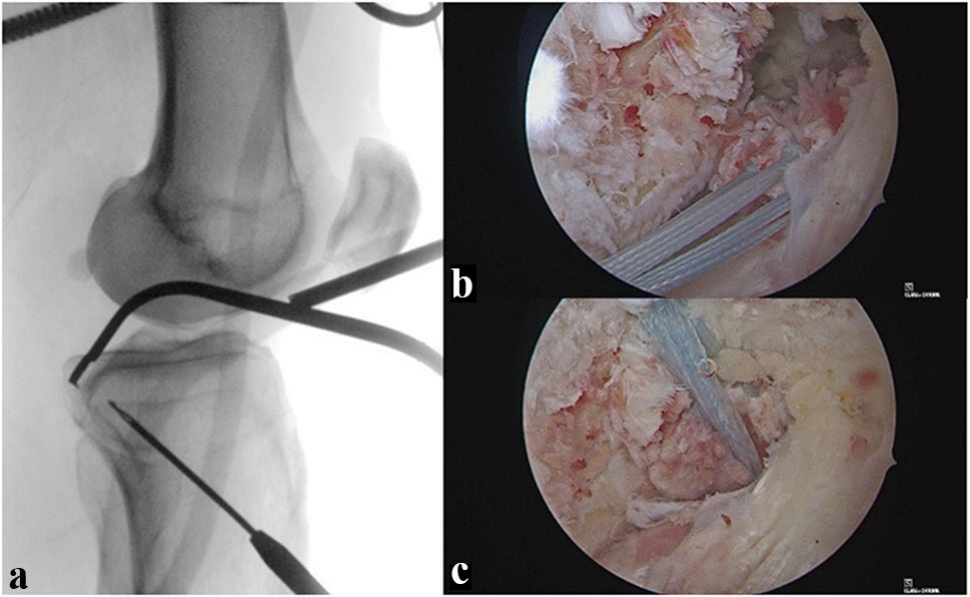
Intraoperative radiographic control of tibial tunnel placement for PCL bracing (**a**). Intraarticular, arthroscopic view with PCL pullout sutures in-situ (**b**) and reattached femoral PCL avulsion with ligament bracing (**c**). *PCL* posterior cruciate ligament

Arthroscopic PCL bracing is then performed (Fig. 2). Depending on the localization and ligament remnants of the PCL lesion, tibial or femoral avulsions can be addressed by additional pull-out sutures, intraligamentous tears are treated by PCL bracing only. The technique has already been described in context of the treatment of femoral avulsions of the PCL [17] and knee dislocations [11, 13].

Surgical repair of all torn PMC or PLC structures is performed by an additional medial or lateral open surgical approach, after PCL bracing in order to reduce the risk of a fixed posterior translation [27]. Intraligamentous tears are treated by direct end-to-end suturing, tibial/ fibular or femoral avulsions are refixed by suture anchors.

**Fig. 3:**
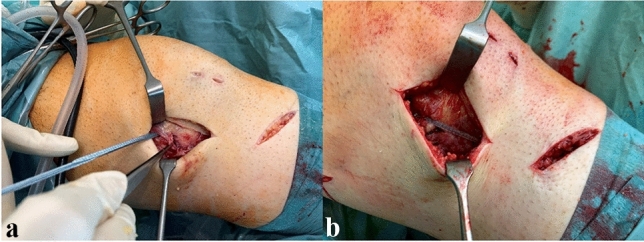
PMC injury with femoral MCL avulsion with prepared anchor at the site of femoral MCL avulsion (**a**). Final result after femoral MCL refixation and additional sMCL/POL bracing (**b**). PMC posteromedial corner. (s)MCL (superficial) medial collateral ligament. *POL* posterior oblique ligament

On the medial site, the superficial (sMCL) and deep (dMCL) medial collateral ligament as well as the posterior oblique ligament (POL) are addressed separately, on the lateral site the lateral collateral ligament (LCL), popliteus tendon (PT), biceps femoris tendon and posterolateral capsule, respectively. Depending on the severity and extend of injury and the preference of the surgeon, posteromedial injuries are braced with non-resorbable tape in the course of the sMCL and POL according to the technique described by LaPrade et al. [28] (Fig. 3).

**Fig. 4:**
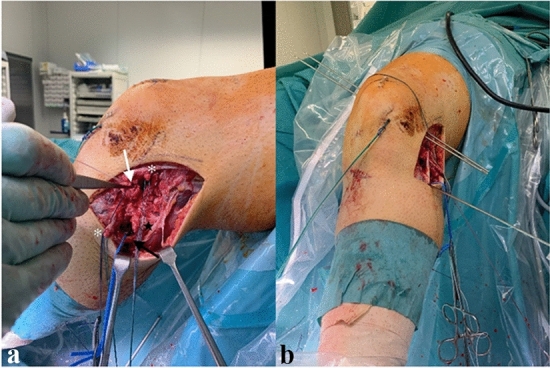
Management of PLC injury (**a**) with securing of the peroneal nerve (black arrow), prepared side-to-side sutures of an intraligamentous LCL tear (white star), popliteal tendon (white arrow) as well as biceps femoris tendon avulsion (black star). Drill pins inserted for primary posterolateral ligament augmentation with semitendinosus autograft (**b**). *PLC* posterolateral corner; *LCL* lateral collateral ligament

For PLC injuries, additional primary ligament augmentation with a semitendinosus autograft is conducted depending on extend and severity of injury or surgeon’s preference. The applied techniques of posterolateral ligament augmentation have been described by Arciero et al. [29] (Fig. 4) and Larson et al. [30].

### Rehabilitation

The rehabilitation itself is determined by the presence of concomitant intraarticular pathologies, and therefore was adapted individually. In general, patients with ligament bracing of the PCL as well as sMCL/POL on the one hand and primary PLC ligament augmentation on the other hand are allowed to fulfil a relatively progressive rehabilitation protocol due to the high primary stability of the constructs. Range of motion (ROM) is limited to extension/flexion 0/0/60° for four weeks, followed by 0/0/90° for another two weeks. Patients are mobilized under partial weight bearing with 20 kg over six weeks. A stabilizing brace is prescribed for the first six weeks. Posterior tibial supporting braces are not necessary.

### Follow-up

For functional outcome measurement, the International Knee Documentation Committee (IKDC) subjective questionnaire and objective examination form [31], Lysholm Score [32, 33], pre- and postoperative Tegner Activity Scale [32, 33] as well as the Knee Injury and Osteoarthritis Outcome Score (KOOS) were queried.

Side-to-side differences in posterior translation were quantified by standardized routine stress-radiographs (Telos®; Wölfersheim, Germany) of both knees with 15 kp posterior tibial force in 90° of flexion at the time of follow-up examination.

Accompanying meniscal and chondral injuries, peri- and postoperative complications and surgical revisions were documented.

### Statistical analysis

Findings are reported by the mean value for continuous data (standard deviation between parentheses) and number for categorical data (percentage between parentheses). T-tests for continuous variables were performed between the subgroups PMC versus PLC. All tests were two-sided, and statistical significance was set at p < 0.05. Analyses were performed using IBM SPSS Statistics version 27 software (IBM Corp., Armonk, New York).

## Results

N = 33 patients with combined PCL injuries were initially included in the study. Two patients (6.1%) did not attend the follow up examination and were lost to follow-up, so that n = 31 (female-to-male ratio = 7:24; mean age 39.1 ± 13.8 years, range 18–60 years) were available for statistical analysis.

The mean time interval between injury and operation was 8.5 ± 3.7 days, the follow-up examinations were performed on average 16.8 ± 9.6 months after the operation.

Regarding the PCL injury, five patients (15.6%) had tibial PCL avulsions and eight (25.8%) femoral PCL avulsions, whereas the majority (18, 58.4%) suffered from intraligamentous PCL tears.

The PMC group consisted of 18 patients versus 13 patients in the PLC group.

Additional intraarticular pathologies were found in a relevant number of cases: In 32.2% diagnostic arthroscopy revealed meniscal tears, of which 70% involved the medial meniscus, 20% the lateral meniscus and 10% both menisci. Six patients (19.4%) were proven to have traumatic chondral lesions grade III-IV according to the International Cartilage Regeneration & Joint Preservation Society (ICRS).

Accompanying vascular injuries were not documented, two patients showed a posttraumatic neural impairment in terms of transient peroneal nerve palsies with spontaneous remission until follow-up.

Regarding the operative treatment, all patients included received arthroscopic ligament bracing of the PCL, in 54.8% additional transosseous PCL suture repair was needed.

In the PMC group, eleven patients were treated by suture repair only, seven received additional ligament bracing. In the PLC group, isolated posterolateral suture repair was performed in five (22.6%), whereas additional primary posterolateral ligament augmentation by semitendinosus autograft was conducted in eight (19.3%) cases, respectively.

Basic demographic data as well as injury characteristics are shown in Table 1.

**Table 1:** Study population

Participating centres		6 (5 GER; 1 SUI)
Included		33
Lost to follow-up		2
Sex		
	Female	7
	Male	24
Age		39.1 (range 18–60) years

		n (%)
PCL injury		31
	Tibial	5 (15.6)
	Femoral	8 (25.8)
	Intraligamentous	18 (58.4)
PMC		
	Tibial	4 (12.9)
	Femoral	9 (29)
	Intraligamentous	5 (16.1)
	Intact	13 (41.9)
PLC		
	Fibular	2 (6.5)
	Femoral	3 (9.7)
	Intraligamentous	8 (25.8)
	Intact	18 (58)
Meniscus injury		
	Medial	7 (22.6)
	Lateral	2 (6.5)
	Medial + lateral	1 (3.2)
	Intact	21 (67.7)
Cartilage injury		
	Grade III-IV	6 (19.4)
	Intact	25 (80.6)

*PCL* posterior cruciate ligament; *PMC* posteromedial corner; *PLC* posterolateral corner

Postoperative complications included one infection (3.2%), four cases (12.9%) with knee stiffness and three cases (9.7%) of symptomatic postoperative instability. All cases presenting with complications needed revision surgery, the overall revision rate was 25.8%. Successful debridement and irrigation were performed in the case of wound infection in one step, knee stiffnesses were addressed by arthroscopic arthrolysis, and persistent instabilities were treated by secondary ligament reconstructions. PCL, PMC and PLC treatment modalities and postoperative complications are listed in Table 2.

**Table 2:** Operative treatment and complications

	n (%)
PCL	
Ligament Bracing	14 (45.2)
Ligament Bracing + Suture	17 (54.8)
PMC	
Repair	11 (35.5)
Repair + Ligament Bracing	7 (22.5)
PLC	
Repair	5 (16.1)
Repair + Reconstruction	8 (25.8)
Complications	
Infection	1 (3.2)
Stiffness	4 (12.9)
Instability	3 (9.6)

*PCL* posterior cruciate ligament; *PMC* posteromedial corner; *PLC* posterolateral corner

The mean Lysholm Score for all patients included was 85 ± 14.8 (range 46–100), the KOOS was 89.7 ± 8.1 (range 63–99), respectively. On average, patients lost 0.9 ± 1.3 points (range 0–5) on Tegner Activity Scale between pre-injury level and follow-up examination.

The average IKDC score for all patients was 69.8 ± 16.5 (range 43.7–100). N = 14 (45.2%) patients were graded A (“normal knee function”), n = 8 (25.8%) B (“near normal knee function”), n = 2 C (“abnormal knee function”) and n = 3 D (“severely abnormal knee function”). All patients graded D—considered as failure of treatment – were affiliated to the PLC group and received secondary PCL and posterolateral ligament reconstructions.

Comparing PMC and PLC regarding IKDC, KOOS and Tegner difference, there was a tendency towards more favourable outcomes in the PMC group, as all parameters showed better mean absolute scores compared to the PLC group. Though, these differences were not statistically significant (n.s.).

**Fig. 5:**
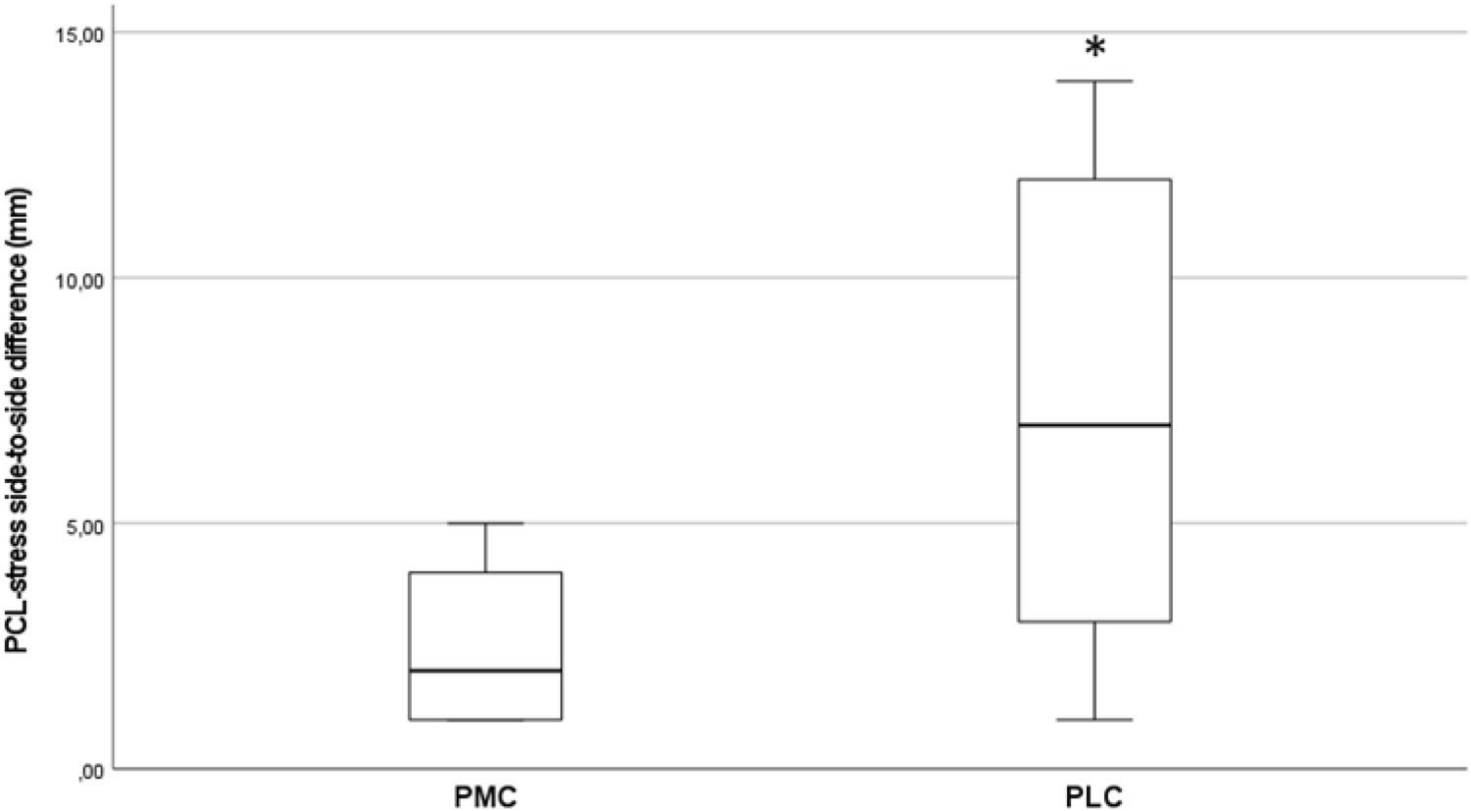
PCL stress-radiography testing, comparing the results of PMC versus PLC. The asterisk (*) indicates a significant difference in comparison at p = 0.035. *PMC* posteromedial corner; *PLC* posterolateral corner

The results of the PCL stress-radiography testing showed an overall side-to-side difference of 3.7±3.8mm. The mean difference in the PMC group was 2.5 ± 1.5mm (range 1–5) versus 5.8 ± 5.6mm (range 0–14) in the PLC group. This difference was statistically significant (p = 0.035; Figure 5).

The overall results for the postoperative functional outcome as well as a separation by PMC and PLC groups are shown in Tables 3 and 4.

**Table 3:** Overall outcome scores at a mean of 16 months postoperative

	Minimum	Maximum	Mean ± SD
Stress radiography (mm)	0	14	3.7 ± 3.8
Tegner pre-op	0	10	5.0 ± 1.9
Tegner post-op	0	7	4.1 ± 1.5
Tegner difference	0	5	0.9 ± 1.3
Lysholm	46	100	85.0 ± 14.4
KOOS	63	99	89.7 ± 8.1
IKDC	43.7	100	69.8 ± 16.5

**Table 4:** Statistical subgroup analysis of acute combined PCL lesions with additional PMC versus PLC injury

	Minimum	Maximum	Mean ± SD	p-value
Stress radiography (mm)				
PMC	1	5	2.5 ± 1.5	0.036
PLC	0	14	5.8 ± 5.6	
Tegner pre-op				
PMC	0	7	4.8 ± 1,8	0.53
PLC	4	10	5.3 ± 2.1	
Tegner post-op				
PMC	0	7	4.3 ± 1.6	0.56
PLC	4	10	3.9 ± 1.2	
Tegner difference				
PMC	0	3	0.6 ± 0.9	0.21
PLC	0	5	1.3 ± 1.7	
Lysholm				
PMC	55	100	87.9 ± 12.1	0.19
PLC	46	93	80.3 ± 17.1	
KOOS				
PMC	63	99	90.4 ± 9.0	0.61
PLC	76.2	97	88.7 ± 6.7	
IKDC				
PMC	43.7	100	72.2 ± 18.9	0.36
PLC	49.4	82	66.0 ± 11.6	

*PMC* posteromedial corner; *PLC* posterolateral corner

## Discussion

The most important finding of this study was that one-stage arthroscopic suture repair and ligament bracing is a safe and viable technique to treat acute combined PCL injuries (PCL plus PMC or PLC) with good to excellent clinical outcomes regarding knee function, stability and activity levels. Patients treated for additional PMC injuries seem to deliver more reliable results regarding knee function and objective stability than patients with PLC injuries. To our knowledge, this is the first study investigating this systematically in a relevant number of patients in a multicentre study setting.

The feasibility of the technique of primary suture repair and PCL bracing has been proven by several authors in the past [17–24]. It was biomechanically shown that isolated suture repair of the PCL can withstand less than 100N load before failure, and that PCL suturing without bracing does not lead to satisfactory clinical results [34, 35]. However, all mentioned studies are only technical notes or case series (n = 2 maximum) describing the operative aspects of primary PCL suture repair and ligament bracing in contrast to primary ligament reconstruction.

In 2020, Vermeijden et al. published a systematic review discussing patient selection, surgical technique, and outcomes of primary repair of PCL tears [36]. A total of eight studies with n = 101 patients were identified reporting on outcome parameters after PCL suturing and optional PCL bracing. If documented, most studies present the results of femoral avulsion injuries (91–100% of the cases), only one study focusses on tibial avulsion injuries [37]. In contrast to this the recent study population contains all tear characteristics with 15.6% tibial PCL avulsions, 25.8% femoral avulsions and 58.4% intraligamentous tears which are not necessarily applicable for pullout sutures. Subsequently, in 45.2% PCL bracing without suturing was performed. None of the studies included in the review article evaluated objective knee laxity by side-to-side difference PCL stress-radiography. According to Vermeijden et al. the Lysholm score ranged 85.2–96.7, the loss of activity (Tegner) 1.1–2.2 points and the IKDC score was 75.7–94. These satisfying results a comparable to the results of this examination. Though, the IKDC showed worse outcomes with 69.8 ± 16.5 in the recent study. This may be explained by more severe injury characteristics of the patient population in the present study, as in the review by Vermeijden a relevant number of patients with PCL tears without documented presence of concomitant collateral ligament injuries were included. Consequently, better functional outcome scores can be expected due to the lower grade of instability [5].

Recently, Hopper et al. investigated the two-year outcome of PCL repair with suture augmentation of n = 17 patients in one of the rare prospective case series available [38]. Indications included acute grade III PCL tears, but also symptomatic chronic tears and PCL tears as part of multiligamentous knee injuries. The authors report satisfactory patient reported outcome measures, including a mean KOOS of 87.0. All patients were found to have a stable knee on manual clinical examination. The results this study match to the satisfactory findings by Hopper et al., as similar KOOS and PCL stress-radiography values are reported. Unfortunately, Hopper et al. do not differentiate acute from chronic and isolated from combined PCL injuries in particular in their study, so that the two investigations are not comparable without restrictions.

Otto et al. reported on a case series of n = 14 patients with acute PCL lesions in multiple injured knees surgically treated with internal bracing with a mean follow-up of 19.9 ± 7.7 months [14]. The surgical technique of PCL bracing is similar to the one described here. However, the case series is heterogeneous regarding the injury characteristics, as 57% of patients presented with knee dislocations type III to V according to Schenck et al. [39] and 43% involving the PMC and/or PLC. The PMC and PLC groups are not evaluated separately regarding the outcome measurements. The authors found a mean Lysholm Score of 69.1 ± 16.6 and a mean IKDC of 68.9 ± 18.1, respectively. Additionally, the knee stability was rated by PCL stress-radiography showing a mean side-to-side difference of 5.5 ± 4.1 mm. The authors summarize that their surgical technique leads to adequate restoration of posterior tibial translation. This is confirmed by the recent study results, as similar IKDC scores could be reached, and outcomes that are even more favourable regarding Lysholm scores and radiographic posterior tibial translation.

Due to the lack of literature concerning combined PCL injuries in particular, the study results must be related to the available results of surgical treatment of knee dislocations with inhomogeneous patient populations. In literature, both one-stage and two-stage procedures were described. A well-established two-stage approach contains surgical stabilization of medial or lateral peripheral ligament structures and initial conservative treatment of the PCL injury by external bracing. According to literature, failure rates of 20% with remaining chronic PCL instability must be expected [10, 40, 41], and should be addressed by secondary PCL ligament reconstruction. Alternatively, a one-stage hybrid surgical treatment can be conducted by initial suture repair of the periphery and simultaneous PCL reconstruction with a tendon autograft [26].

In 2019, a prospective multicentre study on the outcome of early surgical repair with additional ligament bracing of all torn ligaments in acute knee dislocations was published by Heitmann et al. [13]. N = 73 patients with acute knee dislocations and a mean follow-up of 14 ± 1.6 months could be included. Regarding functional outcome, the mean IKDC was 75.5 ± 14.5, Lysholm score was 81.0 ± 15.5, and loss of activity (Tegner) was 1 (range 0–3) point. PCL stress-radiographs showed side-to-side differences at a mean of 2.9 ± 2.1 mm. These overall good to excellent results could be reproduced in this study. The surgical technique applied in the recent study population is equivalent to the one described for the treatment of knee dislocations, but inclusion criteria are different as the recent stud study focusses on designated PCL injuries with additional PMC or PLC injuries, excluding knee dislocations. The overall revision rate was found to be higher (25.8%) than in the population evaluated by Heitmann et al. (17.4%), but only 9.7% of the cases needed revision surgery due to persistent knee instability. The most common indications for revision in the study of Heitmann et al. were stiffness and instability, leading to re-operations in 14.5% of the cases. Interestingly, the rate of concomitant chondral and meniscal injuries was high in both studies with 46.4% and 52.0%, respectively. Deducting data from the available literature, it can be hypothesized that the mid- to long-term outcome may also be determined by the high rate of accompanying intraarticular pathologies [42].

Evaluating the subgroups of PMC and PLC in the recent study, we found a significant difference regarding objective knee stability in PCL stress-radiographies. All outcome measurements for knee function and activity level (IKDC, KOOS, Tegner difference) showed a tendency towards more favourable outcomes of PMC, and all failures of treatment—defined as indication for secondary ligament reconstruction—were affiliated to the PLC group. Unfortunately, literature comparing functional results of surgical treatment of combined PCL injuries, differentiating PMC versus PLC injuries is lacking.

In 2019, Chahla et al. published an expert consensus statement regarding diagnosis and treatment of knee ligament injuries involving the PLC [9]. In the setting of acute injuries, a combination of repair and ligament augmentation is recommended. In advance, different other authors could demonstrate that isolated suture repair of the PLC results in inferior functional outcomes compared to ligament augmentation techniques [43, 44]. Though, these investigations have been performed in the context of knee dislocations or isolated PLC injuries without additional PLC injury. An advantage of primary ligament reconstruction in the setting of combined PCL injuries versus PLC suture repair only could not be proved in this study due to small subgroups and should be evaluated in further studies with more patients included.

On the medial side of the knee, it is widely accepted that MCL tears in context with multiligamentous knee injuries require surgical treatment, whereas low-grade isolated injuries can be managed conservatively. Therapeutic options in the acute setting include suture repair of all torn structures and optional ligament bracing or primary reconstruction [5, 45–47]. Recently, another international expert consensus statement on diagnosis and treatment of injuries to the PMC was published [48]. In general, previous recommendations regarding their treatment are confirmed. Interestingly, experts agreed (80% consensus rate) that for combined PCL injuries involving the PMC it is reasonable to treat these injuries conservatively with a dynamic PCL brace. Though, the authors did not differentiate high grade tears (Grade II and III according to [25]) with significant clinical instability from partial ligament tears. Contrary to this, excellent results could be reached in here by PCL suture repair and PCL bracing as well as open surgical repair with or without additional bracing or ligament augmentation of the PMC.

The major strength of this study is that it is the first investigation of acute combined PCL injuries treated with one-stage arthroscopic surgical repair and ligament bracing in a larger cohort. Nevertheless, this specific injury combination is rarely seen. Therefore, a prospective multicentre study was conducted accompanied with its typical limitations (different surgeons, different clinical settings). Results are presented only in the short-term follow-up. Another limitation was the size of the study population so that subgroup analysis could not be performed. The lack of significance comparing PMC and PLC may be explained by too little statistical power. That is why investigations with larger study populations comparing PMC and PLC injuries as well as suture repair / refixation versus primary ligament augmentation by autograft within the PLC group are desirable.

## Conclusions

One-stage suture repair and ligament bracing seem to be a safe and viable technique to treat acute combined PLC injuries involving the PMC or PLC with good to excellent clinical outcomes. Though, patients with PLC injuries show a tendency towards inferior clinical outcomes and higher instability rates. Complication and revision rates are comparable to its application in knee dislocations. Consequently, the technique of suture repair and ligament bracing in the setting of acute combined PCL injuries involving the PMC or PLC can be recommended based on this first investigation dealing with this issue.

## Data Availability

The data presented in this study are available on reasonable request from the corresponding author.
